# UHF RFID Sensing for Dynamic Tag Detection and Behavior Recognition: A Multi-Feature Analysis and Dual-Path Residual Network Approach

**DOI:** 10.3390/s25175540

**Published:** 2025-09-05

**Authors:** Honggang Wang, Xinyi Liu, Lei Liu, Bo Qin, Ruoyu Pan, Shengli Pang

**Affiliations:** College of Communication and Information Engineering, Xi’an University of Posts and Telecommunications, Xi’an 710121, China; wanghonggang@xupt.edu.cn (H.W.); liulei@stu.xupt.edu.cn (L.L.); qinbo@stu.xupt.edu.cn (B.Q.); panruoyu@xupt.edu.cn (R.P.); pangshengli@xupt.edu.cn (S.P.)

**Keywords:** behavior recognition, dynamic tag detection, dual-path residual network, isolated forest, UHF RFID

## Abstract

To address the challenges of dynamic coupling interference and time-frequency feature degradation in current approaches to Ultra-High-Frequency Radio-Frequency Identification (UHF RFID) behavior recognition, this study proposes a novel behavior recognition method integrating multi-feature analysis with a dual-path residual network. The proposed method mitigates interference by using phase difference methods to eliminate signal errors and cross-correlation, as well as adaptive equalization algorithms to decouple interfering signals. To identify the target tags participating in behavioral interactions, we construct a three-dimensional feature space and apply an improved weighted isolated forest algorithm to detect active tags during interactions. Subsequently, Doppler shift analysis extracts behavioral features, and multiscale wavelet-packet decomposition generates time-frequency representations. The dual-path residual network then fuses global and local features from these time-frequency representations for behavioral classification, thereby identifying interaction behaviors such as ‘taking away’, ‘putting back’, and ‘hesitation’ (characterized by picking up, then putting back). Experimental results demonstrate that the proposed scheme achieves behavioral recognition accuracy of 94% in complex scenarios, significantly enhancing the overall robustness of interaction behavior recognition.

## 1. Introduction

Human behavior recognition, as one of the core technologies of pervasive computing, plays a crucial role in transforming service models in fields such as smart homes, medical monitoring, and retail [1,2,3]. Compared with traditional computer vision solutions, which suffer from issues such as light sensitivity and privacy leaks [4], RFID-based technology offers significant advantages, owing to its properties of non-contact sensing and strong environmental adaptability. In particular, device-free sensing methods [5,6,7,8,9,10] achieve behavior recognition by analyzing electromagnetic field disturbances caused by RFID tags deployed in the environment, thereby avoiding user compliance issues associated with wearable devices.

Existing RFID behavior recognition technology has undergone two evolutionary phases: early wearable solutions require fixed tags on the human body [11,12], leading to limited usability; later device-free methods, while freeing users from physical constraints [13], still face significant challenges, particularly with respect to signal interference. In particular, in scenarios with dense tag deployment, accurately identifying specific tags that interact with the human body is a critical challenge.

This paper proposes a novel behavior recognition method for ultra-high-frequency passive RFID systems, aiming to address the technical challenges of severe multipath interference and inaccurate target identification caused by densely distributed tags in crowded environments. The method establishes a recognition framework based on a dual-path residual network with multi-feature fusion. The processing pipeline begins with preprocessing pf the raw phase and RSSI signals using an autocorrelation algorithm and an adaptive filter to effectively suppress multipath effects. Subsequently, an improved isolation forest algorithm is employed to automatically screen and identify dynamic tags. Finally, wavelet transform and the dual-path network are integrated to deeply fuse spatiotemporal features, achieving high-precision and highly robust behavior recognition in complex scenarios. This study provides an effective and scalable technical solution for typical applications such as intelligent security and warehouse management.

**Figure 1:**
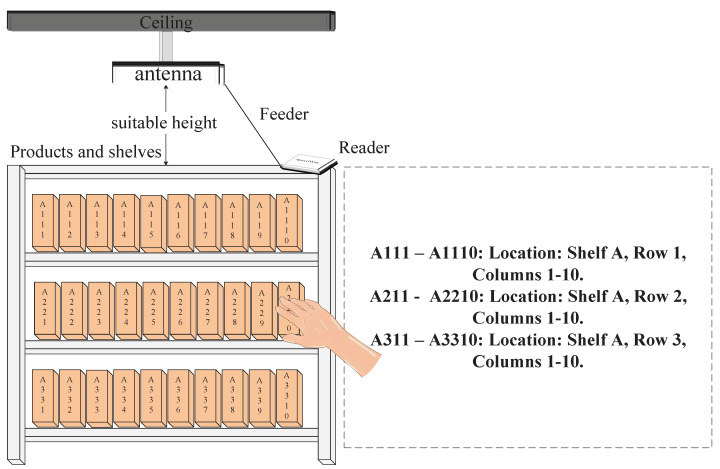
Schematic diagram of UHF RFID tags and antenna layout scenarios in the shelf area.

The research scenario is illustrated in Figure 1. By attaching RFID tags to the sides of shelf items and optimizing antenna installation positions slightly above the shelf to avoid obstruction, the system achieves precise recognition of user interaction behaviors such as ‘take-away’, ‘put-back’, and ‘hesitation’.

The main contributions of this system are summarized below.

1. We develop a diversity suppression model to reduce interference with the signal from tag and human characteristics.
2. We construct a 3D feature space incorporating the IQR temporal fluctuation index, cumulative phase difference, and tag-state evaluation metrics, combined with an improved isolation forest algorithm, to achieve precise dynamic tag detection.
3. We develop a behavior recognition model to segment behavior streams and use Doppler frequency analysis to identify specific behavior characteristics and changes. We also construct a time-frequency map based on wavelet transform to illustrate energy changes in behavior and propose a dual-path residual network to recognize behaviors such as taking away, putting back, picking up, and putting back.
4. We develop an optimization engine for marketing strategies in a retail scenario to convert behavior recognition results into actionable business decisions.

The remainder of this paper is organized as follows. Section 2 introduces related work. Section 3 introduces an overview of the proposed system. Section 4 introduces the specific implementation of the system. Section 5 proposes marketing strategies for human pattern analysis and comprehensively evaluates the performance of the proposed system.

## 2. Related Work

Related research has mainly focused on multipath effect mitigation, Doppler shift-based motion recognition, and deep learning behavior recognition methods.

### 2.1. Autocorrelation Method to Mitigate Multipath Effects

Existing research on RFID often inadequately addresses the multipath effect, resulting in raw signals (such as phase and RSSI) containing substantial noise caused by environmental and human reflections, which leads to a low signal-to-noise ratio and directly compromises the accuracy of subsequent analysis. Multipath effects are a common challenge in wireless communications, and mature autocorrelation suppression techniques have been developed in the GNSS field. Reference [14] proposed a multipath suppression technique based on a partial autocorrelation function to suppress short-delay multipath signals. Reference [15] focused on multi-GNSS research, proposed a unified parameter suppression method based on autocorrelation, and verified its effectiveness in terms of the correlation between different GNSS frequencies and the validity of the unified parameters. These methods provide important insights for RFID research. On the one hand, partial autocorrelation techniques can be directly applied to suppress short-delay interference; on the other hand, the correlation between multi-frequency RFID systems can be explored to develop unified parameter multipath suppression schemes, thereby simplifying signal processing workflows in multi-frequency environments.

### 2.2. Behavior Recognition Based on Doppler Frequency Shift

Doppler frequency-shift curves are of significant value in human behavior analysis, as they can capture shifts in signal frequency caused by human movement, thereby distinguishing between different actions/behaviors. For example, RF-RES [16] uses Doppler frequency shift to track chest displacement caused by breathing; RFPass [17] captures Doppler frequency shift and utilizes gait features for user authentication; Tag-Fall [18] combines Doppler frequency shift, velocity, and position information to determine falls; FEMO [19] uses Doppler frequency shift to identify free-weight exercise types; CBID [20] uses the Doppler effect to detect tag movement for shopping behavior recognition; the method proposed by Chen et al. [21] uses RFID tags attached to key skeletal nodes to identify various human activities through Doppler frequency shift, RSSI, and phase information; and FGSA [22] uses Doppler frequency shift with dual-tag phase calibration to identify shopping behavior. However, most of the aforementioned studies are limited to ideal scenarios (e.g., with a sparse number of tags or known target tags), making it difficult to automatically identify moving targets in dense tag environments. The dynamic tag detection method proposed in this paper, combined with Doppler shift analysis, enables autonomous screening of moving tags and behavior recognition in complex scenarios, significantly improving the system’s universality and environmental adaptability.

### 2.3. Behavior Recognition Based on Deep Learning

In recent years, deep learning has garnered significant attention in the field of human behavior recognition and has been integrated with classical machine learning to enhance model robustness and generalization capabilities. For example, SATCN [23] combines TCN and self-attention mechanisms to improve the accuracy of RFID-based indoor human behavior recognition; TSCNN [24] uses 3D convolutional neural networks to extract spatio-temporal features from RFID RSSI data for behavior classification; the method proposed in Ref. [25] comprises a non-wearable human motion recognition scheme based on STGCN integrating phase and RSSI data; and the TagSee [26] system uses single-antenna RFID imaging and deep learning to analyze customer blocking signals, enabling contactless customer behavior analysis. Ding et al. [27] validated the effectiveness of multimodal feature fusion in human interaction behavior classification by coupling a 2D-CNN with ResNet. RFnet [28] applies a dual-path structure to RFID temporal signal processing, using the spatial residual path to capture static poses and the temporal residual path to model dynamic evolution. Building upon the aforementioned deep learning approaches, this paper proposes a dual-path residual network-based method for action recognition. By integrating both global and local motion features, it achieves complementary enhancement of motion information, thereby improving the accuracy of action recognition.

## 3. System Overview

**Figure 2:**
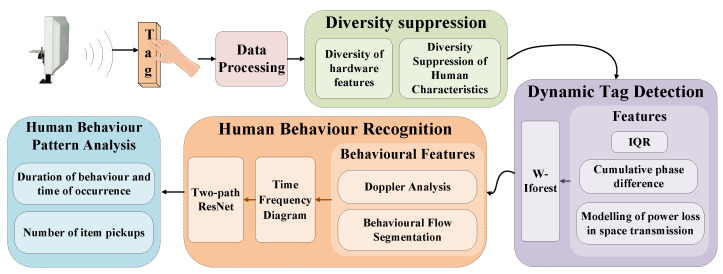
System architecture diagram.

The core research process is shown in Figure 2 and includes the following:

(1) Preprocessing of labeled data using phase unwrapping and smoothing filtering;
(2) Diversity suppression to effectively reduce tag hardware characteristics and environmental and human interference;
(3) Dynamic tag detection, using a three-dimensional feature matrix containing IQR time-domain fluctuations, cumulative phase difference phase-domain dynamics, and tag-state evaluation indicators, combined with an improved isolation forest algorithm for dynamic tag detection;
(4) Behavior recognition utilizing Doppler frequency to analyze behavior characteristics, combined with wavelet transform to generate time-frequency maps of behavior energy features and a dual-path residual network for behavior classification;
(5) Analysis of key indicators, such as the number of times tags are picked up or put back and the duration of behavior, to analyze human behavior patterns.

## 4. System Design

### 4.1. Data Preprocessing

Phase signals have periodic characteristics that can easily lead to phase entanglements during measurement [29]. Therefore, phase disentanglement is used to restore the true phase-change trajectory, which can be achieved by using (1).

(1) $$\phi_{u}=\phi_{w}+2\pi k,$$

where $\phi_{u}$ is the unwrapped phase (rad), $\phi_{w}$ is the wrapped measurement (rad), and k∈Z counts the 2π phase jumps.

**Figure 3:**
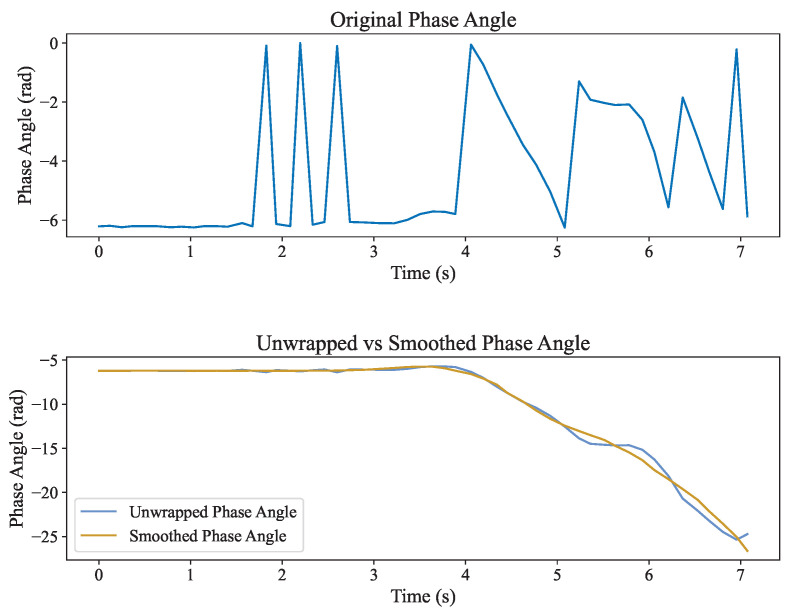
The first plot shows the raw, periodic phase signal. The second shows the unwrapped and smoothed result, revealing the true trajectory with suppressed noise.

As shown in Figure 3, the disentangled phase signal and the simultaneously obtained RSSI signal were filtered and smoothed using a Savitzky–Golay filter to effectively reduce noise.

### 4.2. Feature Diversity Suppression

The hardware characteristics of tags not only determine their sensitivity to the environment but also directly affect the signal transmission efficiency and reliability. Simultaneously, differences in human height and weight can alter signal propagation paths and intensity, affecting signal stability and accuracy.

#### 4.2.1. Tag Hardware Feature Suppression

**Figure 4:**
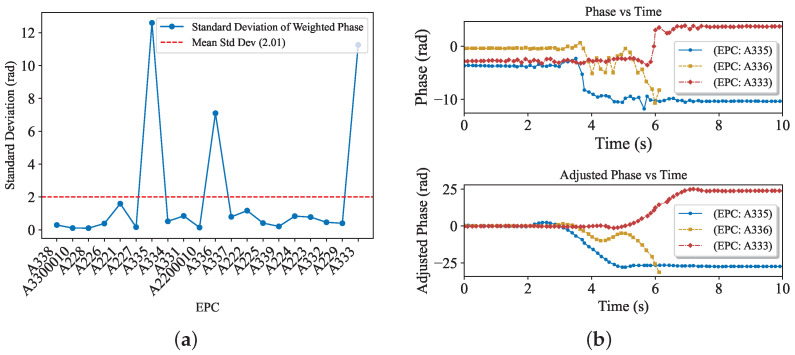
(**a**) The distribution of the standard phase deviation of individual tags at rest in the tag matrix. (**b**) The phase change of the tag after it has been suppressed by the hardware characteristics.

As shown in Figure 4a, although most static tags exhibit stable characteristics, some exhibit abnormal fluctuations. To improve the accuracy of environmental perception, it is necessary to effectively suppress the inherent characteristics of the tags. During the static detection phase, the phase information ($\theta_{s_{ij}}$) of sample *j* of the *i*-th tag includes the influence of environmental factors and tag characteristics.

The RF phase measurement model is expressed as follows:

(2) $$\theta_{s,ij}=\left(\frac{4\pi d_{ij}}{\lambda }+\theta_{\mathrm{tag},ij}+\theta_{e,ij}\right)\;\mathrm{mod}\;2\pi$$

The signal travels a round-trip path (from the reader to the tag and back to the reader), so the actual propagation distance is $2d_{ij}$, resulting in a phase change that is twice that of a one-way trip. Hence, the coefficient is 4π. Here, λ is the signal wavelength, and $d_{ij}$ denotes the distance between the *j*-th sample of the *i*-th tag and the antenna (m). The first part reflects the phase delay of the electromagnetic wave along the propagation path. $\theta_{\mathrm{tag},ij}$ represents the intrinsic phase offset of the tag hardware (rad), and $\theta_{e,ij}$ captures the environmental phase noise with $\theta_{e,ij}∼N\left(0,\sigma^{2}\right)$.

The phase value $\theta_{d,ij}$ of the *j*-th sample of the *i*-th tag during the behavior process includes the characteristics of the environment and tags, as well as the influence of the human hand.

(3) $$\theta_{d,ij}=\left(\frac{4\pi d_{ij}}{\lambda }+\theta_{\mathrm{tag},ij}+\theta_{e,ij}+\theta_{\mathrm{hand},ij}\right)\;\mathrm{mod}\;2\pi$$

where $\theta_{\mathrm{hand},ij}$ is hand-induced phase perturbation (rad).

Environmental and tag-specific influences are eliminated by subtracting the static tag means, yielding the adjusted dynamic phase ($\theta_{d,ij}^{\prime }$).

(4) $$\theta_{d,ij}^{\prime }=\left(\frac{4\pi }{\lambda }\left(d_{ij}-{\tilde{d}}_{i}\right)+\theta_{\mathrm{hand},ij}\right)\;\mathrm{mod}\;2\pi ,\;j\in Z^{+}$$

where ${\tilde{d}}_{i}$ is the distance for the *i*-th tag (m) and $Z^{+}$ is a set of positive integers.

As shown in Figure 4b, the starting points of the processed phase signals are aligned, which greatly improves the comparability of the data and the accuracy of the subsequent analysis.

#### 4.2.2. Suppression of Human Characteristics

**Figure 5:**
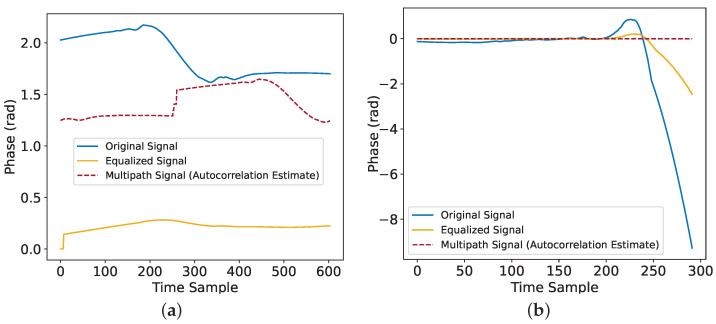
(**a**) Phase change of a static tag after inhibition of human characteristics. (**b**) Phase change of a dynamic tag after human trait suppression.

To address the issue of multipath interference caused by the human body in wireless signals, this study proposes a joint suppression scheme integrating partial autocorrelation estimation (PACE ) and adaptive equalization. The scheme first uses the PACE algorithm to extract the delay and amplitude characteristics of the multipath signals, thereby constructing a multipath interference model and achieving signal reconstruction. Subsequently, a variable-step-length LMS adaptive equalizer is employed to suppress residual interference, and finally, polarity correction is applied to maintain the signal integrity. As shown in Figure 5a, the originally fluctuating signal error of the static tag was mitigated, resulting in a smoother state. As shown in Figure 5b, the phase data of the processed dynamic tag still clearly shows the fluctuation characteristics caused by this behavior.

### 4.3. Dynamic Tag Detection

#### 4.3.1. Volatility Analysis

**Figure 6:**
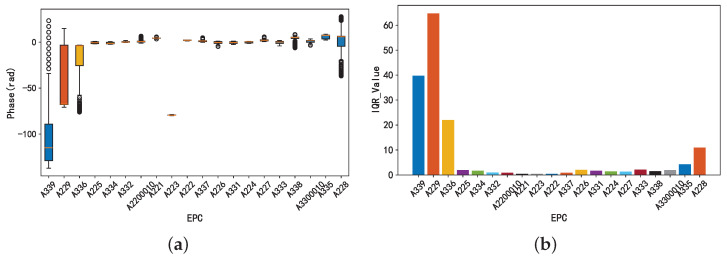
(**a**) Volatility characteristics of different tags. The boxplots display the distribution of phase values (in radians) for each EPC tag. The central box represents the interquartile range (IQR), the horizontal line within the box marks the median, and the whiskers extend to 1.5 × IQR. The circles beyond the whiskers indicate outliers—individual phase readings that deviate significantly from the majority of the data.(**b**) The volatility characteristics of the tag are quantified by IQR values.

After feature diversity suppression, the phase data were unified and aligned to zero, eliminating the original scale differences. However, as shown in the box plot in Figure 6a, the phase data still exhibits significant variability during the behavioral process. To robustly quantify this variability, the study employed the interquartile range (IQR). The IQR provides a more robust measure of the core dispersion of data than the standard deviation, particularly for highly variable phase data containing outliers. As shown in Figure 6b, there were notable differences in the IQR values across different tags. Higher IQR values indicate greater variability in tag data, indicating more pronounced changes in phase features during behavioral processes.

#### 4.3.2. Phase Characteristic Analysis

When a tag is moved, changes in its position cause alterations in the signal propagation path, leading to fluctuations in phase data. Dynamic tags exhibit significant phase variation characteristics. By calculating the cumulative phase difference, the system can amplify the effects of small but continuous or intense but transient phase changes caused by human movement of the tag. Therefore, monitoring the cumulative effects of phase changes can quantify tag movement caused by human operation.

**Figure 7:**
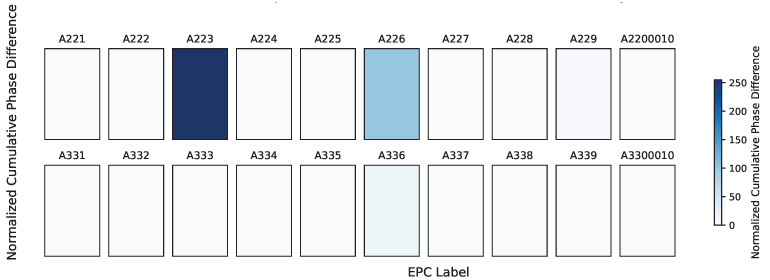
Quantification of the phase change caused by human movement by accumulating phase differences.

The grayscale image of the cumulative phase difference values shown in Figure 7 indicates that the areas of the tag affected by human movement typically exhibit larger cumulative phase difference values. Consequently, the areas of the tag influenced by human movement are clearly depicted in darker colors.

#### 4.3.3. Analysis of the Human Body’s Impact on Signal Characteristics

**Figure 8:**
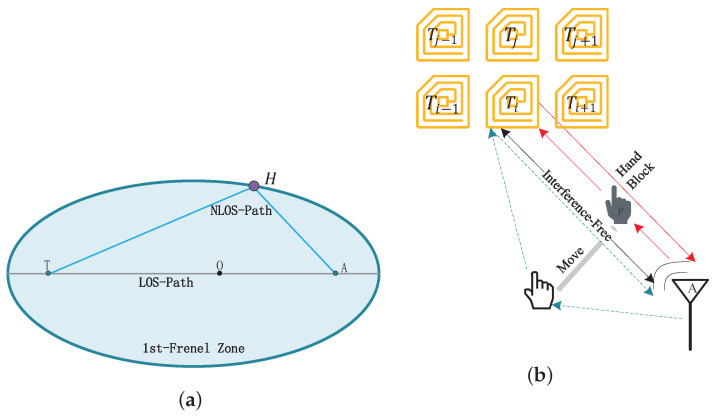
(**a**) The first Fresnel zone model consists of a tag and an antenna. (**b**) A loss model of the power that the tag transmits in space.

In this study, the space between the tag and antenna is modeled as the first Fresnel zone. Under ideal line-of-sight (LOS) propagation conditions, there are no obstacles between the tag and antenna, and signal attenuation is primarily due to free-space loss. In practical applications, the human hand enters the first Fresnel zone, resulting in non-line-of-sight (NLOS) propagation. As shown in Figure 8a, when the hand is located at boundary position H of the first Fresnel zone, the signal emitted by the antenna is blocked by the hand before reaching the tag, resulting in signal attenuation.

Therefore, the actual received signal is the signal blocked by the hand (NLOS) and the signal transmitted by the tag in free space (LOS); thus, the true received signal is $S_{\mathrm{real}}$.

(5) $$S_{\mathrm{real}}=S_{\mathrm{los}}+S_{\mathrm{nlos}}$$

where the $S_{\mathrm{los}}$ component represents free-space propagation and the $S_{\mathrm{nlos}}$ component represents the hand-blocked signal.

Human hand movements can interfere with tag signals. To quantify and analyze their impact on the tag array, a theoretical spatial transmission power loss model was established, as shown in Figure 8b.

Therefore, the LOS signal $S_{{\mathrm{los}}_{i}}$ and NLOS $S_{{\mathrm{nlos}}_{i}}$ signal of the *i*-th tag in the tag array are affected by the hand.

(6) $$S_{\mathrm{los},i}=S_{A\to T_{i}}$$

(7) $$S_{\mathrm{nlos},i}=S_{A\to H}\cdot S_{H\to T_{i}}$$

(8) $$S=\alpha e^{j\theta }$$

where $S_{H\to T_{i}}$ is the signal transmitted directly from the antenna to the *i*-th tag, $S_{A\to H}$ is the signal transmitted from the antenna that is blocked by the hand, $S_{H\to T_{i}}$ represents the signal transmitted to the i-th tag after being obstructed by the hand, and α and θ is channel attenuation and its phase shift.

When the signal is transmitted in free space, assuming there is no other interference (α=1), the phase shift ($\theta_{A\to T_{i}}$) is related to the reader’s operating frequency. Then, $S_{A\to T_{i}}$ is represented as follows:

(9) $$S_{A\to T_{i}}=e^{j\theta_{A\to T_{i}}}$$

In the antenna-to-hand scenario, the hand is treated as an environmental obstacle, and its overall attenuation effect on the signal can be approximated as a constant value (*C*). In the hand-to-tag scenario, the hand dynamically influences the tag, and subtle hand movements can cause significant variations in the tag’s signal data; therefore, the attenuation ($C_{{\mathrm{HT}}_{i}}$) in signal transmission is approximated as $\frac{1}{{\left(d_{{\mathrm{HT}}_{i}}\right)}^{2}}$. $\theta_{\mathrm{AH}}$ represents the phase shift caused by the antenna transmitting to the hand. $\theta_{{\mathrm{HT}}_{i}}$ represents the phase shift caused by the signal returning from hand to tag. Then, $S_{A\to H}$ and $S_{H\to T_{i}}$ are represented as follows:

(10) $$\begin{array}{cc} S_{A\to H} & =C\cdot e^{j\theta_{\mathrm{AH}}} \end{array}$$

(11) $$\begin{array}{cc} S_{H\to T_{i}} & =\frac{1}{d_{{\mathrm{HT}}_{i}}^{2}}e^{j\theta_{{\mathrm{HT}}_{i}}} \end{array}$$

Therefore, the power loss for the *i*-th tag is obtained as follows:

(12) $$P_{\mathrm{hand},i}={\left|S_{A\to H}\cdot S_{H\to T_{i}}\right|}^{2}=\frac{C^{2}}{d_{\mathrm{HT},i}^{4}}$$

In (12), it can be seen that the tag’s reflection power is affected by the distance between the hand and the tag. Because RSSI is a measure of the received power, the likelihood of the tag being picked up can be assessed by comparing the predicted value from the power correlation model with the actual RSSI. The Pearson correlation coefficient (I) was used to quantify the consistency between the two variables.

(13) $$I_{i}=\frac{1}{N_{i}-1}\sum_{j=1}^{N_{i}}\left(\frac{P_{ij}-\mu_{P_{i}}}{\sigma_{P_{i}}}\right)\left(\frac{R_{ij}-\mu_{R_{i}}}{\sigma_{R_{i}}}\right)$$

where $N_{i}$ is the number of samples for a single tag, $P_{i}$ is the power model for the tag, $\mu_{P_{i}}$ and $\mu_{R_{i}}$ are the sample means of $P_{i}$ and $R_{i}$, $\sigma_{P_{i}}$ and $\sigma_{R_{i}}$ are the standard sample deviations of $P_{i}$ and $R_{i}$.

**Figure 9:**
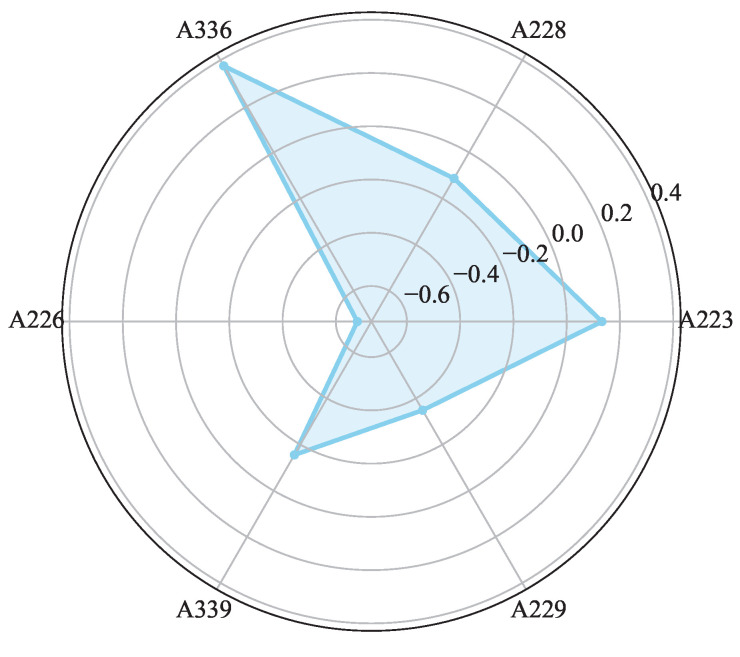
The dynamic possibility of labeling is quantified by constructing a spatial transmission loss model to obtain the correlation between the lost power and the signal strength.

As shown in Figure 9, the probability of picking up different tags was quantified.

#### 4.3.4. Dynamic Tag Detection

Accurately locating dynamic tags in dense RFID environments is essential for passive behavior recognition. To address this, we propose a tag-state evaluation method that integrates multi-feature fusion with an improved isolation forest algorithm. The approach begins by constructing a probabilistic evaluation metric based on a spatial transmission power loss model, combined with time-domain features (IQR and cumulative phase deviation), to achieve quantitative assessment of tag states. This provides high-quality input for the subsequent improved isolation forest process. The feature vector of the *i*-th tag can be represented as $X_{i}$.

(14) $$X_{i}=\left[\begin{array}{ccc} I_{1} & C_{1} & Q_{1}\\ I_{2} & C_{2} & Q_{2}\\ \vdots & \vdots & \vdots \\ I_{N} & C_{N} & Q_{N} \end{array}\right]$$

where $I_{k}$, $C_{k}$, and $Q_{k}$ represent the status evaluation index, cumulative phase difference, and IQR, respectively, for the *k*-th tag.

In the dynamic tag detection phase, $X_{i}$ is used as the input, and the weighted isolated forest (W-IForest) algorithm is employed, which detects dynamic tags as outliers. he pseudocode of this algorithm is presented as Algorithm 1.

The core innovation of this algorithm lies in transforming dynamic tag detection into a weighted anomaly detection problem. By quantifying the ratio of signal dynamic range to volatility and the motion activity level of tags, the algorithm achieves weighted processing of IQR, cumulative phase difference, and signal correlation. During the construction of isolation trees, it prioritizes the splitting of feature dimensions with higher weights. The algorithm calculates anomaly scores through weighted path length, significantly enhancing the detection sensitivity for dynamic tags. This method markedly improves the accuracy and real-time performance of RFID dynamic tag detection in complex environments.
**Algorithm 1:** Weighted Isolation Forest (W-IF).**Require:** Phase signals $\left\{\theta_{i}\right\}$, RSSI $\left\{r_{i}\right\}$, Timestamps $\left\{t_{i}\right\}$ Initial contamination $c_{\mathrm{init}}=0.15$**Ensure:** Anomalous tags $E_{\mathrm{anomaly}}$ with confidence scores1:**for** each tag $EPC_{k}$ **do**2:    $DSR\leftarrow \left(\max\left(r_{i}\right)-\min\left(r_{i}\right)\right)/\left(\mathrm{std}\left(r_{i}\right)+\varepsilon \right)$3:    $\mathrm{motion\_ratio}\leftarrow \sum \left(\mathrm{diff}\left(\theta_{i}\right)>\theta_{\mathrm{thresh}}\right)/\mathrm{len}\left(\theta_{i}\right)$4:    $F_{k}\leftarrow \left[\mathrm{IQR}\left(\theta_{i}\right),\mathrm{cumsum}\left(\mathrm{diff}\left(\theta_{i}\right)\right),\mathrm{corr}\left(\theta_{i},r_{i}\right)\right]$5:    $F_{\mathrm{weighted}}\leftarrow F_{k}⊙\left[0.3\times DSR,0.5\times \mathrm{motion\_ratio},0.2\right]$6:    $w_{\mathrm{sample}}\leftarrow \mathrm{motion\_ratio}\times \mathrm{var}\left(\theta_{i}\right)$7:    $\mathrm{Store}(F_{\mathrm{weighted}},w_{\mathrm{sample}})$8:**end for**9:**for** t=1 to $n_{\mathrm{estimators}}$ **do**10:    $Tree_{t}\leftarrow \mathrm{BuildTree}\left(\mathrm{Features},\mathrm{Weights},\mathrm{strategy}=‘\mathrm{weighted\_gain}’\right)$11:    $\mathrm{AnomalyScores}\leftarrow \mathrm{AnomalyScores}+\mathrm{ScoreSamples}(Tree_{t})$12:    $c_{\mathrm{adj}}\leftarrow c_{\mathrm{init}}\times \left(1+\mathrm{mean}\left(\mathrm{Weights}\right)\right)$13:    $E_{\mathrm{anomaly}}\leftarrow \mathrm{FilterScores}\left(\mathrm{AnomalyScores},\mathrm{percentile}=1-c_{\mathrm{adj}}\right)$14:**end for**15:**return** $E_{\mathrm{anomaly}}$

### 4.4. Behavioral Recognition Model

#### 4.4.1. Behavioral Analysis

**Figure 10:**
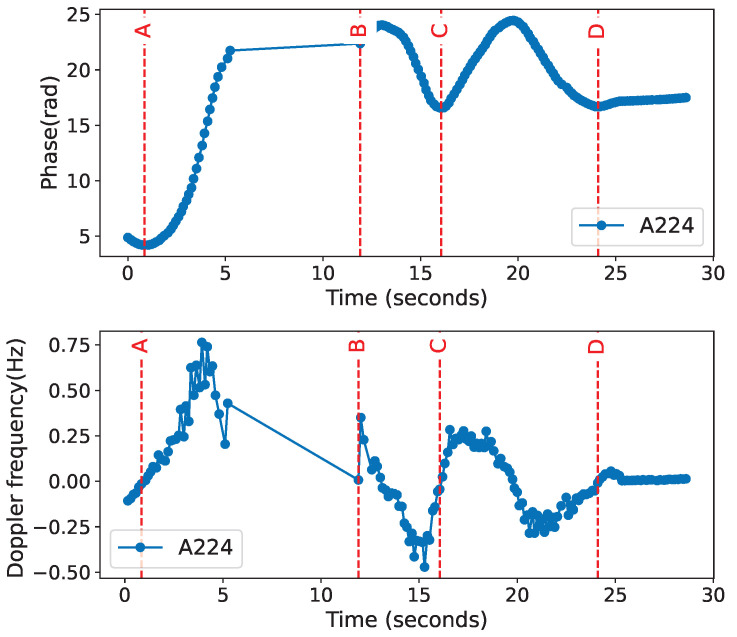
The phase data stream and the Doppler data stream are divided into behavior segments: A–B is take away, B–C is put back, and C–D is pick up and put down.

**Figure 11:**
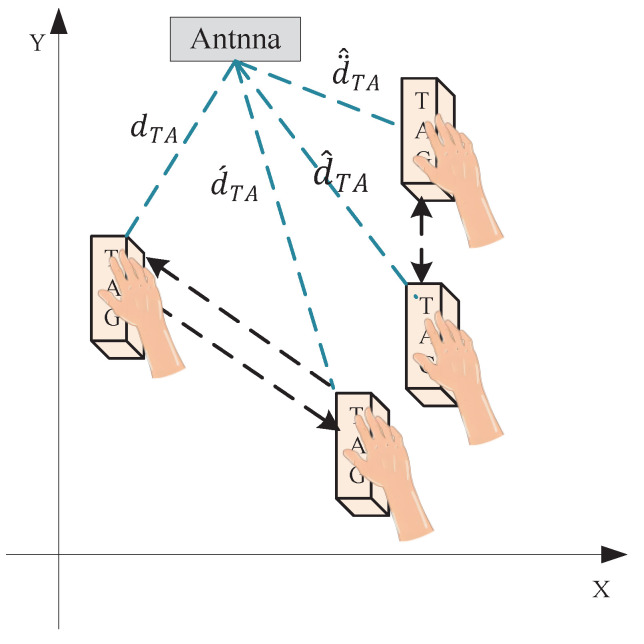
Changes in the relative distance between the tag and the antenna during tag movement.

Following the detection of dynamic tags, the target tags were identified. For accurate analysis of consecutively occurring behaviors, this paper employs a behavior flow segmentation method based on a sliding window. Initially, this method constructs adaptive thresholds by calculating the mean and standard deviation of phase differences within the window, thereby achieving preliminary segmentation. To further enhance the robustness and accuracy of segmentation, the following post-processing steps are introduced: The integration of a minimum amplitude threshold and a minimum duration threshold effectively suppresses noise interference and filters out non-significant instantaneous phase changes. Long-interval detection ensures the completeness of behavior events, preventing premature segmentation due to brief interruptions. Peak point detection precisely captures behavior turning points, such as key moments of picking up or putting down. By synthesizing the aforementioned filtering conditions, continuous phase data is successfully segmented into a series of independent behavior fragments (as depicted in Figure 10). These segmented fragments clearly characterize specific behavior events. Table 1 showcases the specific behavior of each fragment. Based on the properties of the phase, during the process of human movement of the tag, the distance between the tag and the antenna continuously changes, which induces a change in phase, according to (2). Specifically, as illustrated in Figure 11, during the process of the tag being removed from the shelf (assuming the tag moves horizontally away from the antenna along the X-axis at this time), the distance (d) between the tag and the antenna gradually increases, leading to an increase in phase until the tag is moved outside the antenna’s reading range. Conversely, when the tag is put back on the shelf (moving horizontally towards the antenna along the X-axis), the signal re-emerges, and the distance (d) between the tag and the antenna gradually decreases, causing a corresponding change in phase. Therefore, during the process of the tag being horizontally taken away, then put back, the distance between the tag and the antenna undergoes a progression from far to near, followed by a return to stability. For vertical pick-up and put-down actions (e.g., the tag is lifted from a surface, then placed back onto the same surface), although the change in horizontal position may not be significant, the vertical distance between the tag and the antenna experiences a substantial variation. Specifically, when the tag is picked up, the distance between the tag and the antenna decreases, and the phase consequently reduces; when the tag is put down, the distance between the tag and the antenna increases, and the phase correspondingly increases.

To specifically analyze the relative changes between tags and antennas caused by human behavior, this study introduces the Doppler effect as a key analytical tool. The relative motion between the tag and the antenna causes an offset in the signal frequency. This offset in frequency change is known as the Doppler frequency shift. By accurately capturing and quantifying this Doppler frequency shift, we can indirectly reflect the relative motion state between the tag and the antenna, thereby identifying and distinguishing different behavioral patterns.

The Doppler shift arises from the relative motion between the transmitter and receiver. In this scenario, it occurs between a stationary reader and a moving tag. Assuming the reader receives two consecutive signals from the moving tag at times $t_{i}$ and $t_{i+1}$, with phase readings of $\theta_{i}$ and $\theta_{i+1}$, respectively, let *v* represent the tag’s velocity during time interval $\left[t_{i},t_{i+1}\right]$. Since the time interval between consecutive tag readings is very short, *v* can be considered constant. Therefore, the distance (*d*) moved by the tag equals $v\cdot (t_{i+1}-t_{i})$.

In backscatter communication, the signal propagation distance is twice the actual movement distance (2*d*). Combining this with Equation (2), we obtain the following:

(15) $$\lambda \cdot \left(\frac{\theta_{i+1}-\theta_{i}}{2\pi }\right)=2v\cdot \left(t_{i+1}-t_{i}\right)$$

The change in this path difference leads to a corresponding shift in phase, from which the Doppler frequency shift can be derived by analyzing the rate of phase change over time. The quantification of the Doppler effect is presented in (16).

(16) $$f=\frac{\nu }{\lambda }=\frac{\Delta {\tilde{\theta }}_{ij}}{4\pi \cdot \Delta t_{ij}}$$

where $\Delta {\tilde{\theta }}_{ij}$ is the phase difference of the *i*-th tag after suppression of tag diversity and $\Delta t_{ij}$ is the time difference of the *i*-th tag.

**Figure 12:**
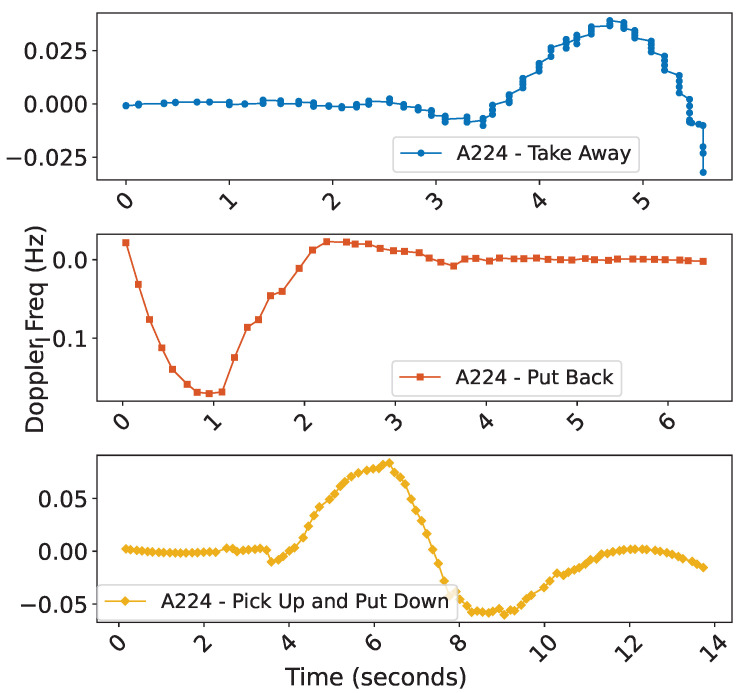
The estimated Doppler frequencies are used to analyze the characteristics of the three behaviors in detail.

Therefore, by utilizing the Doppler frequency variation over time, we can obtain the relative changes between the tag and the reader and analyze behavioral characteristics.Three primary motion behavior patterns can be identified. As illustrated in Figure 12, the tag starts moving at point A. The patterns of take away” and “put back” and the combined actions of “pick up” and “put down” all exhibit unique and distinguishable change characteristics.

Compared to the traditional Doppler behavior analysis method introduced in Section 2, which can only analyze known static tags in a controlled environment, the analysis technology proposed in this study combining dynamic tag detection with Doppler has significant advantages: it can not only accurately identify target tags and achieve precise behavior analysis but also work stably in complex environments with dense tags. Through the aforementioned tag detection algorithm, the system can distinguish between static and dynamic tags, effectively filter environmental interference, and significantly enhance its applicability and reliability in real-world scenarios.

#### 4.4.2. CWT-Frequency Analysis of Behavioral Characteristics

**Figure 13:**
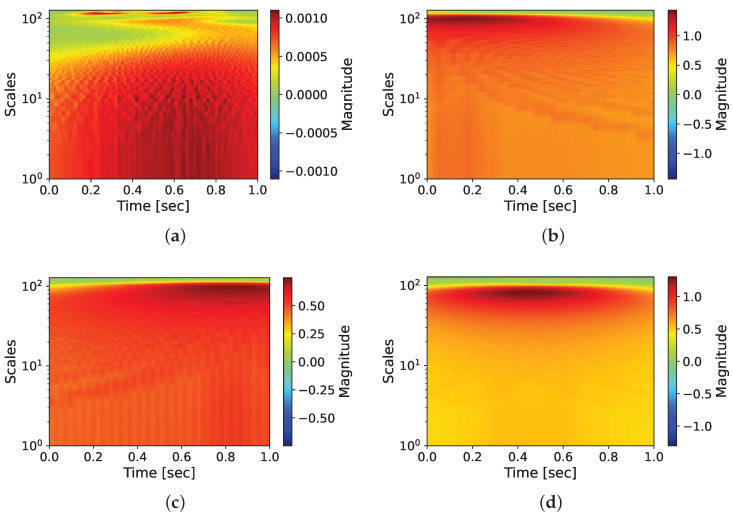
Time-frequency plot of continuous wavelet transform based on Morlet wavelet: (**a**) Stationary. (**b**) Put back. (**c**) Take away. (**d**) Pick up and put down.

To illustrate behavioral characteristics more intuitively, this study a employs continuous wavelet transform (CWT) combined with Morlet wavelet basis functions for time-frequency feature extraction of Doppler signals. By precisely calculating the actual frequency range of the signal, an appropriate scale sequence is constructed, and a time-frequency plot is generated that clearly illustrates the process of signal energy variation over time. The plot shown in Figure 13 visually represents the distribution of the signal energy through color changes, with darker regions corresponding to the occurrence of actions or changes in intensity, effectively revealing the dynamic characteristics within the behavioral segmentation intervals. The intensity of time-frequency energy directly reflects the degree of change in behavioral characteristics, providing a visual basis for action analysis.

#### 4.4.3. Behavior Recognition Model Based on Dual-Path Residual Network

**Figure 14:**
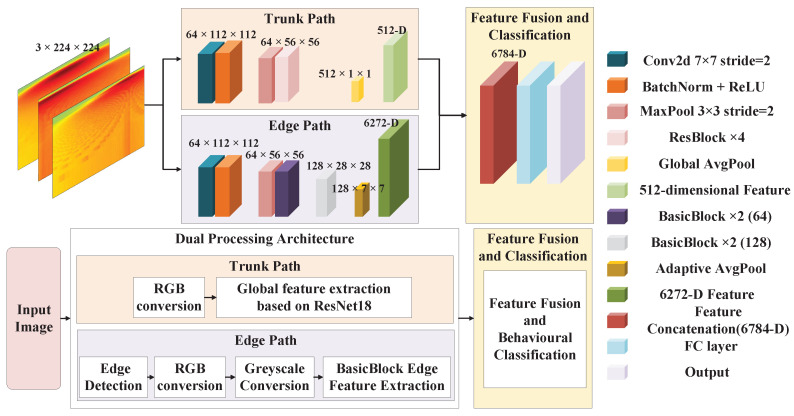
Dual-path residual network architecture.

To achieve behavior recognition, this study employs the Dual-Path Res-Net model to enable end-to-end learning and recognition of behavioral features. As shown in the model diagram in Figure 14, the proposed model adopts a dual-modal parallel processing architecture.

**Main Path:** This path focuses on extracting the global spectral features of the behavior. By processing the wavelet time-frequency map using ResNet18 and extracting 512-dimensional global spectral features through four residual blocks, it captures macro patterns in the time-frequency domain.

**Edge Path:** This path focuses on capturing the local dynamic edge information of the behaviors. We applied adaptive edge detection to preprocessed data (grayscale/Gaussian filtering) and extracted local dynamic features using a lightweight CNN.

**Feature Fusion and Classification:** The features extracted from both paths were concatenated in the fusion layer to form a 6784-dimensional comprehensive feature vector. This vector was then input into the fully connected layer to predict the behavior categories.

**Model Optimization and Training:** To optimize the model’s performance, this study employs an SGD optimizer combined with cross-entropy loss for training, utilizing hyperparameter search and early stopping mechanisms to optimize the model, ultimately achieving high-precision end-to-end behavior recognition.

## 5. System Implementation and Evaluation

This section proposes marketing strategies tailored for human behavior pattern analysis, with a comprehensive experimental evaluation conducted to assess performance.

### 5.1. Human Behavior Pattern Analysis

This study breaks through the limitations of traditional sales data analysis by accurately recording key indicators such as the number of interactions between customers and goods, the duration of interactions, and interaction behavior, thereby constructing a multi-dimensional behavioral analysis system. This method not only enables real-time tracking of changes in the status of goods on shelves but also captures behavioral characteristics in the customer decision-making process with respect to purchasing, providing more comprehensive and accurate data support for retail behavior analysis.

**Figure 15:**
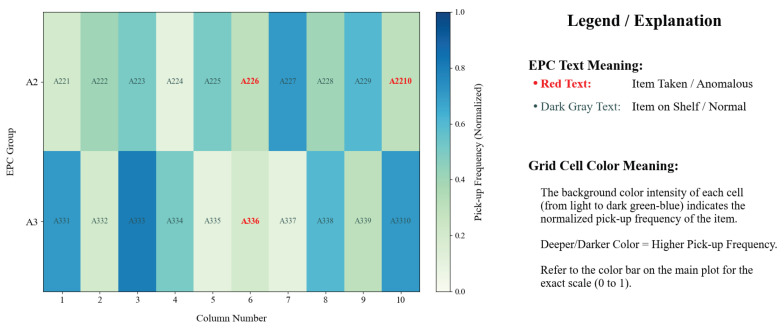
Pick-up count monitoring with shelf status visualization.

Based on the statistics shown in Figure 15 and Table 2, regarding the number of times products were picked up and their seasonal changes from January to June, retailers can adopt a refined strategy: for products with high pick-up frequencies and strong sales performance, they should increase inventory levels and place them in core promotional areas to ensure supply and maximize sales; for products with lower pick-up frequencies and poor performance, they should employ diversified promotional tactics (such as discounts) and re-plan their shelf locations, making them more visible and accessible to enhance exposure and stimulate purchases, ultimately achieving efficient store resource allocation and improved sales performance.

**Figure 16:**
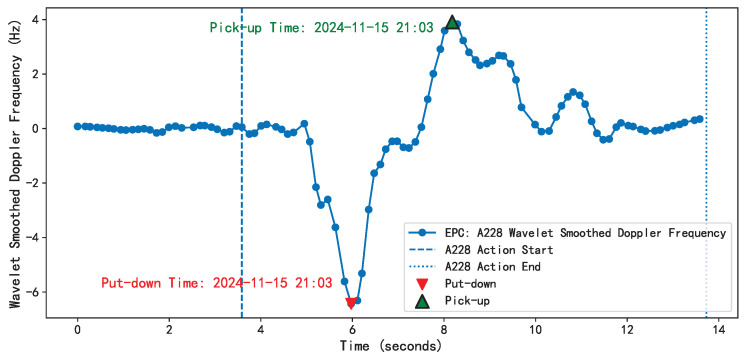
Time identification of behavioral events: Doppler frequency after wavelet smoothing with a time range of 0–14 s. The red triangle indicates the drop action, and the green triangle indicates the pick-up action. The dotted line marks the start and end time points of the behavior, respectively.

The study precisely identifies the moment when customers pick up and put back items, indicating the start and end of the behavior. As shown in Figure 16, on 15 November 2024, at 21:03:39, a certain tag was put down, and at 21:03:42, the tag was picked up, indicating that the customer interacted with the product for three seconds, thereby quantifying the customer’s interest in the product.

By analyzing customers’ contact frequency with products, holding duration, and behavioral sequences, this method deeply decodes product attractiveness and purchase-decision barriers. High-frequency, brief interactions indicate price sensitivity or hesitation in the product style. Such behavioral characteristics can guide retailers to optimize merchandise strategies (price adjustments and style iterations). This approach provides a quantifiable decision-making basis for consumer behavior analysis.

### 5.2. Experimental Evaluation

#### 5.2.1. Experimental Setup

This study was conducted using a COTS UHF Impinj R420 RFID reader and multiple AZ-9662 tags, operating at a fixed frequency of 920.625 MHz and compatible with the EPC Global C1G2 standard, using a circularly polarized antenna with dimensions of 25 × 25 cm and a gain of 9 dBi. When the reader queries the deployed tags, information including the EPC, timestamp, channel frequency, antenna ID, RSSI value, and phase value is transmitted to the host computer via Ethernet. Data is read using Item Test software and statistically analyzed using Python software. The experimental parameters are set as shown in Table 3.

**Figure 17:**
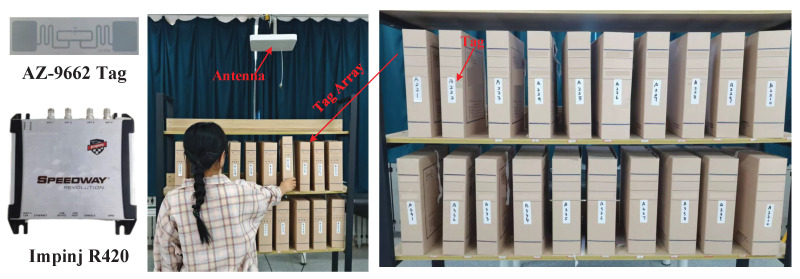
Experimental scenario of the system.

An experimental setup was constructed to validate the performance of the algorithm, as shown in Figure 17. The antennas were deployed at an appropriate height above the shelves to ensure effective coverage and a comprehensive reading of all tags on the shelves. Ten items with tags were placed on the second and third layers of the shelves, with tags affixed to the sides of the items. In the experiment, a human subject entered the area formed by the antennas and tags in any manner and performed actions such as taking away, putting back, picking up, and putting down.

#### 5.2.2. Evaluation of Experimental Results

**Figure 18:**
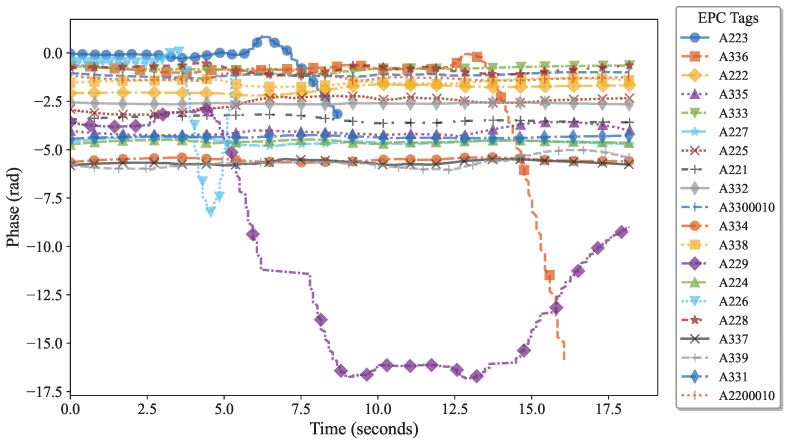
The results of the original phase signal after only phase unwrapping.

To evaluate the robustness of the algorithm in handling human body diversity, this study designed and conducted a user experiment. Three volunteers with distinct body types were recruited to perform interactive behaviors, following their natural habits while reader data was collected. The raw data, after only phase unwrapping, is shown in Figure 18. It can be observed that the initial phase points vary significantly across different volunteers, and adjacent tags (such as A229 and A225) exhibit abnormal fluctuations, which severely interfere with the interpretation of the target interactive behavior.

**Figure 19:**
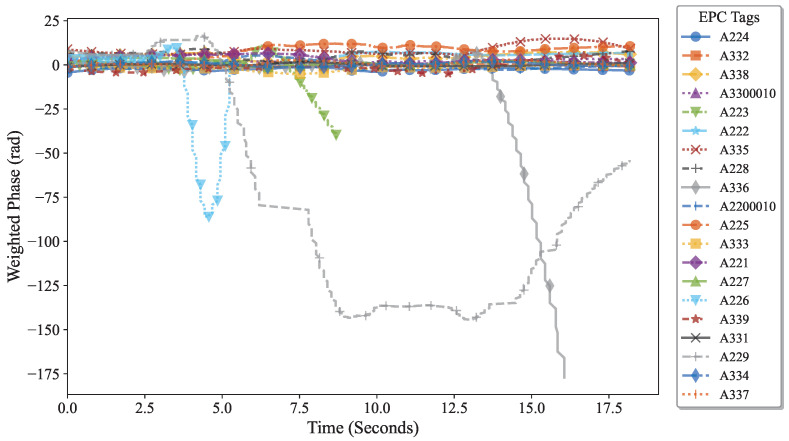
Phase change of the tag array after inhibition by tag diversity.

**Figure 20:**
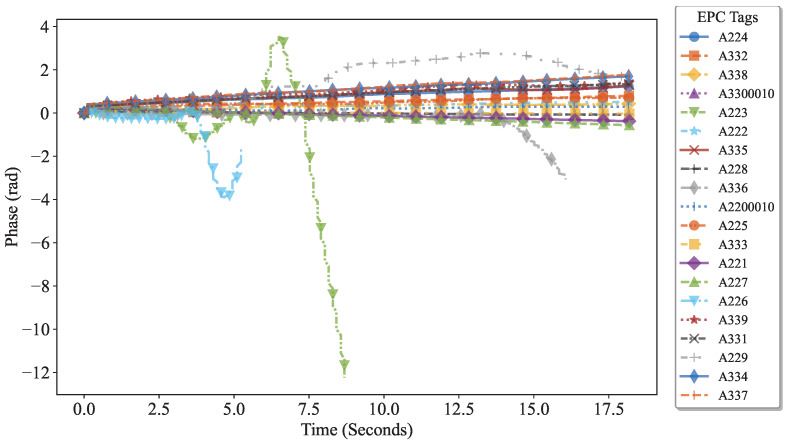
Phase change after inhibition of human diversity.

Therefore, the previously described diversity suppression method was applied to process the data: first, hardware calibration was used to unify the initial phase, eliminating baseline differences and the effects caused by tag hardware characteristics, as shown in Figure 19; then, to address interference from nearby tags due to hand movements, a human feature-based suppression algorithm was employed, effectively highlighting the phase variation patterns of the target tags (A226, A336, and A223). The processed signals shown in Figure 20 significantly reduce the difficulty of target tag identification and provide a clear and reliable data foundation for subsequent interactive behavior analysis. The experimental results verify that the algorithm maintains high accuracy and stability across users with different body types.

Subsequently, as shown in Figure 20, to address interference from hand movements associated with adjacent tags(A229/A225, etc.), a human-body characteristic suppression algorithm was applied. This approach effectively enhanced the distinctive phase variations of the target tags (A226, A336, and A223), significantly facilitating their accurate identification.

**Figure 21:**
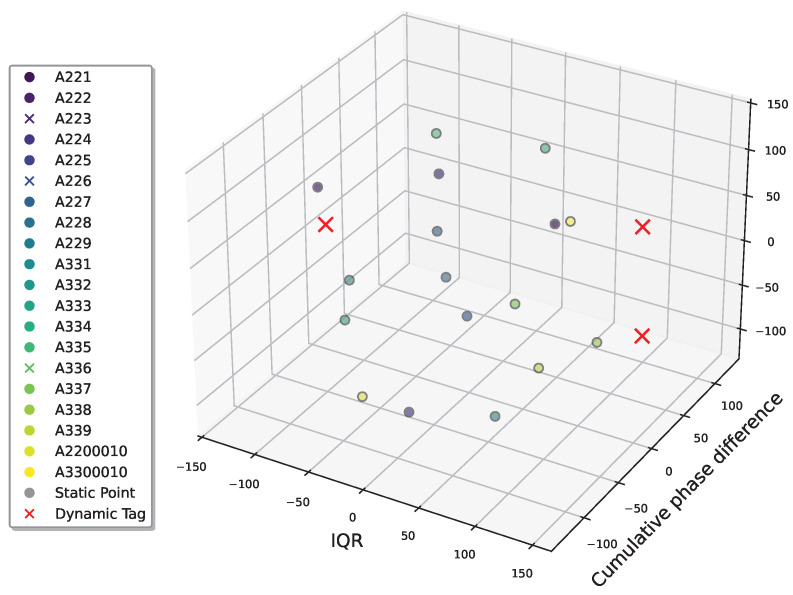
Dynamic tag detection results through isolated forests: the red “x” indicates the detected dynamic tags. “o” indicates normal tags.

This study input the optimized three-dimensional feature matrix into an improved weighted isolated forest model to identify A226, A336, and A223 as dynamic tags (Figure 21), where the red “×” mark indicates a dynamic tag and the “o” mark indicates a static tag.

**Figure 22:**
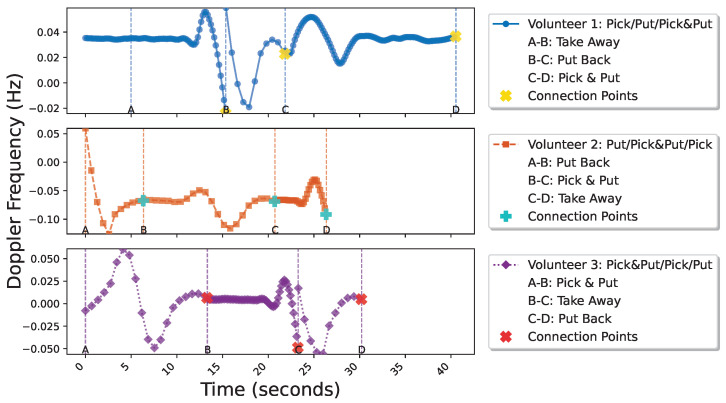
Analysis and segmentation of distinct behavior streams executed by three volunteers at different times and through varying methods.

Based on this, combined with Doppler frequency shift analysis, the experiment accurately captured the Doppler feature changes caused by the interactive behaviors of the three volunteers at different time points, as shown in Figure 22, with the behavior flow of the volunteers segmented into corresponding action segments.

The Doppler-based behavioral analysis methods described in the FEMO [19] and ShopMiner [13] literature typically depend on data analysis of specific target tags. In contrast, our proposed system implements tag diversity suppression and dynamic tag detection mechanisms, enabling effective recognition and analysis of arbitrary pick-up or put-down actions without prior knowledge of tag states. This breakthrough provides a more flexible and universal solution for fine-grained behavioral perception in unconstrained scenarios.

Finally, to validate the effectiveness of the proposed Dual-Path Res-Net in behavior recognition tasks, an experimental dataset was constructed using data collected from 30 participants with different physical characteristics (height, weight, and behavioral habits), yielding a total of 900 complete behavior flow samples covering three typical types of shelf interaction behaviors.

**Figure 23:**
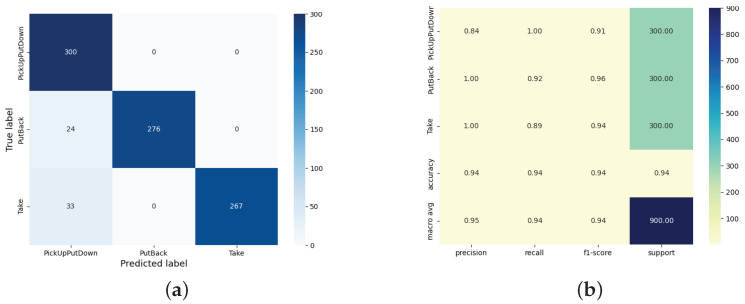
(**a**) Heatmap classification report based on behavior recognition of dual-path residual networks. (**b**) Behavior identification confusion matrix of a two-path residual network. The dark-blue area (diagonal) of the matrix shows the correct classification of the three behavior categories.

As shown in Figure 23a, this model exhibits outstanding performance in recognizing three types of actions, achieving an overall accuracy of 0.94 and an F1 score between 0.94 and 0.95. Among these, the F1 scores for the actions of putting back and taking away both exceed 0.94, and the accuracy for the actions of picking up and putting down is 1.00, although the recall rate is slightly lower (0.84). Figure 23b indicates that the primary misclassifications involve taking away being incorrectly classified as picking up and putting down, along with some misclassification of the putting-back action. This is due to inaccurate boundary determination in behavior segmentation, which leads to distortion of the input data. Segmentation errors not only affect endpoint localization but also decrease the effective training samples for picking up and putting down, resulting in a lower recall rate and increased classification confusion. Future work will focus on optimizing the segmentation algorithm, particularly improving boundary determination accuracy and segmentation robustness.

**Figure 24:**
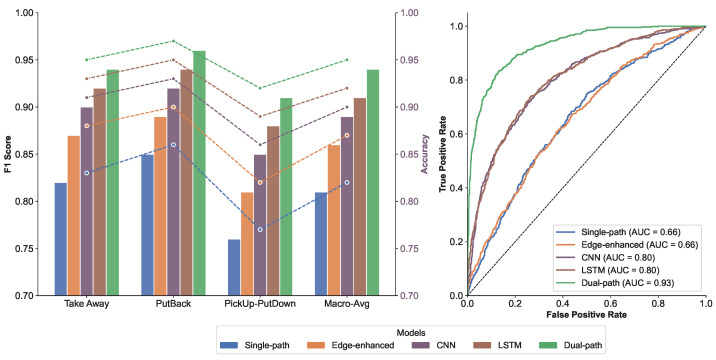
Performance Comparison of Behavior Recognition Models: The left subplot compares evaluation metrics across different models, with the bar chart representing accuracy and the line graph corresponding to the F1 score. The results demonstrate that the Dual-ResNet model outperforms other methods on both metrics. The right subplot shows the ROC curves of various models, where Dual-ResNet achieves an AUC value of 0.983, significantly higher than those of the SVM and CNN models, further confirming its superior classification capability.

This study validates the superior performance of the dual-path residual network in behavior recognition through comparative experiments. As shown in the left subplot of Figure 24, the model achieves a macro-averaged F1 score of 0.94 (an 8.2%improvement over the single-path model) and an accuracy of 95%(a 5.3%improvement compared to the CNN baseline). The right subplot of Figure 24 presents the ROC curve, which demonstrates an AUC value of 0.93 and maintains a true-positive rate of 89%at a false-positive rate of 0.2. By synergistically integrating global spectral features and local edge information through its residual and attention pathways, the model achieves an interaction recognition accuracy of 95%while reducing the false-positive rate by 40%under challenging conditions (FPR < 0.3).

## 6. Conclusions

This study proposes a novel UHF RFID-based behavior recognition method integrating dual-path residual networks with multi-feature fusion. Addressing the challenge of the vulnerability of UHF RFID signals to interference, this research innovatively employs static baseline subtraction, PACE analysis, and adaptive equalization techniques to effectively suppress environmental noise, human-body interference, and multipath effects. An improved isolation forest model accurately detects dynamic tags, whereas Doppler shift analysis captures behavioral characteristics by converting the acquired time-frequency information into wavelet time-frequency maps. The dual-path residual network subsequently performs deep feature learning, significantly enhancing recognition performance through the fusion of global spectral and local edge features. Experimental validation with 30 participants demonstrated the system’s reliability and practicality, achieving 95% recognition accuracy with an F1 score of 0.94. This technology provides an innovative solution for implicit user behavior perceptions in smart retail and intelligent warehousing applications.

However, faced with more complex behavioral combinations and contextual constraints in real retail environments, this study still has the following limitations and corresponding future research directions:

The current method performs well in recognizing simple continuous behaviors, but its temporal action segmentation capability remains insufficient when dealing with complex continuous, combined, and interactive behaviors. Future work could introduce more powerful temporal modeling mechanisms to effectively capture long-range dependencies between actions. Secondly, existing experiments were conducted in controlled environments, whereas real retail scenarios often involve multiple customers moving simultaneously, frequent occlusions, and device signal interference. Subsequent research should validate system robustness in open and dynamic environments and explore strategies such as adversarial training or meta-learning to enhance model generalization. Furthermore, although the current method can recognize macro-level behaviors (e.g., picking up or placing goods), it struggles to distinguish between fine-grained actions with similar intentions (such as “shopping” vs. “stealing” or “organizing” vs. “searching”). Future efforts should incorporate contextual scene information and causal reasoning methods to improve the discrimination and interpretability of behavioral semantics. Finally, although RFID technology offers the advantages of non-visual perception and privacy protection, its limited sensing dimensions restrict the depth of complex behavior understanding. Future research could explore the integration of RFID with low-cost infrared, acoustic, or sparse visual sensors to construct cross-modal behavioral cognition models while maintaining privacy protection, thereby enhancing overall perceptual capabilities.

In summary, while this study has achieved promising results within its defined scope, further advancements are needed in areas such as complex behavior modeling, generalization to open environments, fine-grained behavioral semantic understanding, system optimization, and multimodal fusion to promote the widespread application and implementation of RFID-based behavioral sensing technology in real retail scenarios.

## Figures and Tables

**Table 1:** Behavioral flow segmentation explanation.

Split Interval	Behavior
A–B	Take away
B–C	Put back
C–D	Pick up and put down

**Table 2:** Monthly pick-up counts of retail items (January to June).

Product	January	February	March	April	May	June
Thermos cup	480	450	380	250	180	150
Glass cup	250	260	280	320	380	420
Towel	300	310	320	350	380	450
Umbrella	100	150	350	380	300	250
Flip-flops	50	80	150	300	450	550
Cotton slippers	500	400	200	80	30	10
Shirt	350	380	450	520	600	650

**Table 3:** Reader parameter settings.

Parameter	Value
Reader Type	Impinj Speedway
Tag Type	AZ-9662
Tag Size	70 mm × 17 mm
Protocol	ISO-18000-6C [30]
Reader Read Power	26 dBm

## Data Availability

The data supporting the results reported in this study are not publicly available due to privacy restrictions.
